# Genome-wide (over)view on the actions of vitamin D

**DOI:** 10.3389/fphys.2014.00167

**Published:** 2014-04-29

**Authors:** Carsten Carlberg

**Affiliations:** School of Medicine, Institute of Biomedicine, University of Eastern FinlandKuopio, Finland

**Keywords:** vitamin D, vitamin D receptor, chromatin, gene regulation, epigenomics, genomics

## Abstract

For a global understanding of the physiological impact of the nuclear hormone 1α,25-dihydroxyvitamin D_3_ (1,25(OH)_2_D_3_) the analysis of the genome-wide locations of its high affinity receptor, the transcription factor vitamin D receptor (VDR), is essential. Chromatin immunoprecipitation sequencing (ChIP-seq) in GM10855 and GM10861 lymphoblastoid cells, undifferentiated and lipopolysaccharide-differentiated THP-1 monocytes, LS180 colorectal cancer cells and LX2 hepatic stellate cells revealed between 1000 and 13,000 VDR-specific genomic binding sites. The harmonized analysis of these ChIP-seq datasets indicates that the mechanistic basis for the action of the VDR is independent of the cell type. Formaldehyde-assisted isolation of regulatory elements sequencing (FAIRE-seq) data highlight accessible chromatin regions, which are under control of 1,25(OH)_2_D_3_. In addition, public data, such as from the ENCODE project, allow to relate the genome-wide actions of VDR and 1,25(OH)_2_D_3_ to those of other proteins within the nucleus. For example, locations of the insulator protein CTCF suggest a segregation of the human genome into chromatin domains, of which more than 1000 contain at least one VDR binding site. The integration of all these genome-wide data facilitates the identification of the most important VDR binding sites and associated primary 1,25(OH)_2_D_3_ target genes. Expression changes of these key genes can serve as biomarkers for the actions of vitamin D_3_ and its metabolites in different tissues and cell types of human individuals. Analysis of primary tissues obtained from vitamin D_3_ intervention studies using such markers indicated a large inter-individual variation for the efficiency of vitamin D_3_ supplementation. In conclusion, a genome-wide (over)view on the genomic locations of VDR provides a broader basis for addressing vitamin D's role in health and disease.

## Introduction

During evolution the secosteroid vitamin D_3_ became a pleiotropic signaling molecule (Jones et al., 1998). Initially, the molecule was used by early unicellular organisms to protect their DNA against UV-B irradiation (Holick, 2011). Far later, when the first fish with bones evolved, the endocrinology of vitamin D_3_ was established, and still is very conserved in all higher organisms, including humans (Bouillon and Suda, 2014). In this system, the energy of UV-B is used to convert 7-dehydrocholesterol into pre-vitamin D_3_, i.e., UV-B became essential for the synthesis of vitamin D_3_ (Holick, 2004) (more details in the article by Reichrath et al. in this issue). The central importance of this step is emphasized by the step-wise depigmentation of human skin, when modern humans started to move out of Africa some 100,000 years ago (Hochberg and Templeton, 2010). Two hydroxylation steps are necessary for the conversion of vitamin D_3_ via 25-hydroxyvitamin D_3_ (25(OH)D_3_) into the biologically active vitamin D_3_ metabolite, 1,25(OH)_2_D_3_ (Norman, 2008). The latter molecule participates in a large number of physiological processes, such as bone formation, immune function and cellular growth and differentiation (Deluca, 2004) (more details in the articles by van Leeuwen et al., Hewison et al. and Munoz et al. in this issue).

The transcription factor VDR is the only high-affinity target for 1,25(OH)_2_D_3_ within the cell nucleus (Haussler et al., 1997). VDR is one of approximately 1900 transcription factors, which are encoded by the human genome (Vaquerizas et al., 2009). In addition, VDR is a member of the superfamily of nuclear receptors, most of which are specifically activated by lipophilic molecules in the size of cholesterol (Carlberg and Molnár, 2012). Its lipophilic allows 1,25(OH)_2_D_3_ to pass through all biological membranes, i.e., gene regulation by vitamin D does not involve additional signal transduction steps, as they are known for hydrophilic signaling molecules, such as peptide hormones, growth factors and cytokines. Moreover, VDR is rather ubiquitously expressed, i.e., most human tissues and cell types are responsive to 1,25(OH)_2_D_3_ (Wang et al., 2012).

VDR shares the main structural characteristics of nuclear receptors, which is a highly conserved DNA-binding domain and a structurally conserved ligand-binding domain (Mangelsdorf et al., 1995). VDR's DNA-binding domain specifically contacts the hexameric consensus sequence RGKTSA (R = A or G, K = G or T, S = C or G) within the major groove of genomic DNA (Shaffer and Gewirth, 2002). However, like most other transcription factors, VDR uses a partner DNA-binding protein, in order to bind efficiently to its target sites. More than 20 years ago, this heterodimeric partner turned out to be the nuclear receptor retinoid X receptor (RXR) (Sone et al., 1991; Carlberg et al., 1993). Steric constraints of the dimerizing DNA-binding domains of VDR and RXR determine the optimal binding site of the VDR-RXR complex as a direct repeat of two hexameric nuclear receptor binding motifs spaced by three nucleotides (DR3) (Umesono et al., 1991; Shaffer and Gewirth, 2004). Within VDR's ligand-binding domain, a network of some 40 mostly non-polar amino acids forms a ligand-binding pocket, in which 1,25(OH)_2_D_3_ and its synthetic analogs are specifically fixed with high affinity (Molnár et al., 2006). This ligand binding process induces a conformational change to the surface of VDR's ligand-binding domain, which results in a significant change of VDR's protein-protein interaction profile: it transforms from a repressor to an activator (Moras and Gronemeyer, 1998; Carlberg and Campbell, 2013) (more details on VDR structure in the article by Molnar in this issue).

Taken together, vitamin D signaling primarily comprises the molecular actions of the VDR, i.e., the physiological effects of 1,25(OH)_2_D_3_ are largely identical to those of its receptor. This reduces vitamin D signaling to one central question: which are the most important genomic targets of VDR in a given tissue and which genes are controlled via these sites? Thus, this review focuses on the description of the genome-wide binding of VDR and its mechanistic implications. This analysis will be in the context of genome-wide information on chromatin accessibility and the presence of other nuclear proteins, such as provided by the ENCODE consortium.

## Genome-wide VDR binding

The method chromatin immunoprecipitation (ChIP) was developed, in order to monitor the binding of transcription factors to their genomic targets (Orlando, 2000). The core of the method is (i) mild chemical cross-linking of living cells or tissues, e.g., with 1% formaldehyde, in order to fix nuclear proteins to genomic DNA, (ii) sonication of chromatin into small (200–400 bp) fragments, and (iii) immunoprecipitation with an antibody specific for the chosen nuclear protein (Maston et al., 2012). In this way, chromatin regions, which, at the moment of cross-linking, had been in contact with the protein of choice, are specifically enriched. A specific ChIP signal, in reference to a control (often unspecific IgGs), is a strong indication that the protein of choice had been in contact with the selected genomic region at the moment of cross-linking.

At earlier times, the isolated chromatin template was analyzed by site-specific quantitative PCR (ChIP-qPCR). This approach had been used to study, for example, the extended promoter regions of the primary VDR target genes *CYP24A1* (Väisänen et al., 2005), *CYP27B1* (Turunen et al., 2007), *CCNC* (Sinkkonen et al., 2005), and *CDKN1A* (Saramäki et al., 2006, 2009). Alternatively, the abundance of immunoprecipitated chromatin fragments had been detected by tiled microarrays (so-called “chips,”) which covered a selection of promoter and enhancer regions or any other subset of the genome (ChIP-chip). The group of Pike et al. had extensively used ChIP-chip, in order to locate VDR binding sites within the regulatory regions of the mouse genes *Vdr* (Zella et al., 2006), *Trpv6* (Meyer et al., 2006), *Lrp5* (Fretz et al., 2007), *Tnfsf11* (also known as *Rankl)* (Kim et al., 2006), *Cyp24a1* (Meyer et al., 2010), and *Cbs* (Kriebitzsch et al., 2011). The latest development of the ChIP method is the unbiased analysis of the precipitated chromatin by massively parallel DNA sequencing (ChIP-seq), i.e., the detection of the binding sites of the transcription factor of choice in the complete genome. To date, ChIP-qPCR is primarily used for the confirmation of ChIP-seq results, while ChIP-chip got outdated shortly after its introduction. This leaves, at present, ChIP-seq as the method of choice for analyzing VDR's genomic binding loci.

At present, the readouts of massive parallel sequencing are small sequence tags (35–50 nucleotides), but in the future there will be in majority longer reads used, which will lead to improved significance of the results. These sequence tags are aligned to a reference genome (for human samples this is, at present, hg19) and specifically represent the enriched chromatin fragments. Then “peak calling” software is used to identify genomic regions, in which significantly more sequence tags are detected than in control reactions. Therefore, tags that accumulate as “peaks” at specific genomic loci mark the presence of the investigated nuclear protein (Park, 2009; Furey, 2012). At present, ChIP is still performed with millions of cells; in case of a prominent binding site, most of these cells contribute to the ChIP signal, i.e., it can be assumed that in the majority of cells the locus is occupied by VDR. However, when only in some cells a site is bound by VDR, the respective peak is far less prominent, i.e., most likely of less impact for the regulation of 1,25(OH)_2_D_3_ target genes.

To date, VDR ChIP-seq data are available from (i) the immortalized lymphoblastoid cell lines GM10855 and GM10861 (Ramagopalan et al., 2010), (ii) undifferentiated THP-1 monocyte-like cells (Heikkinen et al., 2011), (iii) lipopolysaccharide (LPS)-polarized THP-1 macrophage-like cells (Tuoresmäki et al., 2014), (iv) LS180 colorectal cancer cells (Meyer et al., 2012), and (v) LX2 hepatic stellate cells (Ding et al., 2013). The original publications reported between 1600 and 6200 VDR binding sites (in ligand-stimulated samples) within the human genome. However, these numbers are not directly comparable, since different peak calling software, alternative threshold settings and even an older version of the reference genome (hg18) were used. A harmonized re-analysis of all six VDR ChIP-seq datasets with identical peak calling settings (MACS, version 2) resulted for 1,25(OH)_2_D_3_-stimulated and unstimulated cells, respectively, in following number of binding sites: 6172 and 3144 (GM10855), 12,353 and 4072 (GM10861), 774 and 609 (undifferentiated THP-1), 953 and 529 (LPS-differentiated THP-1), 3777 and 165 (LS180) and 1532 and 1474 (LX2) (Tuoresmäki et al., 2014).

**Figure 1:**
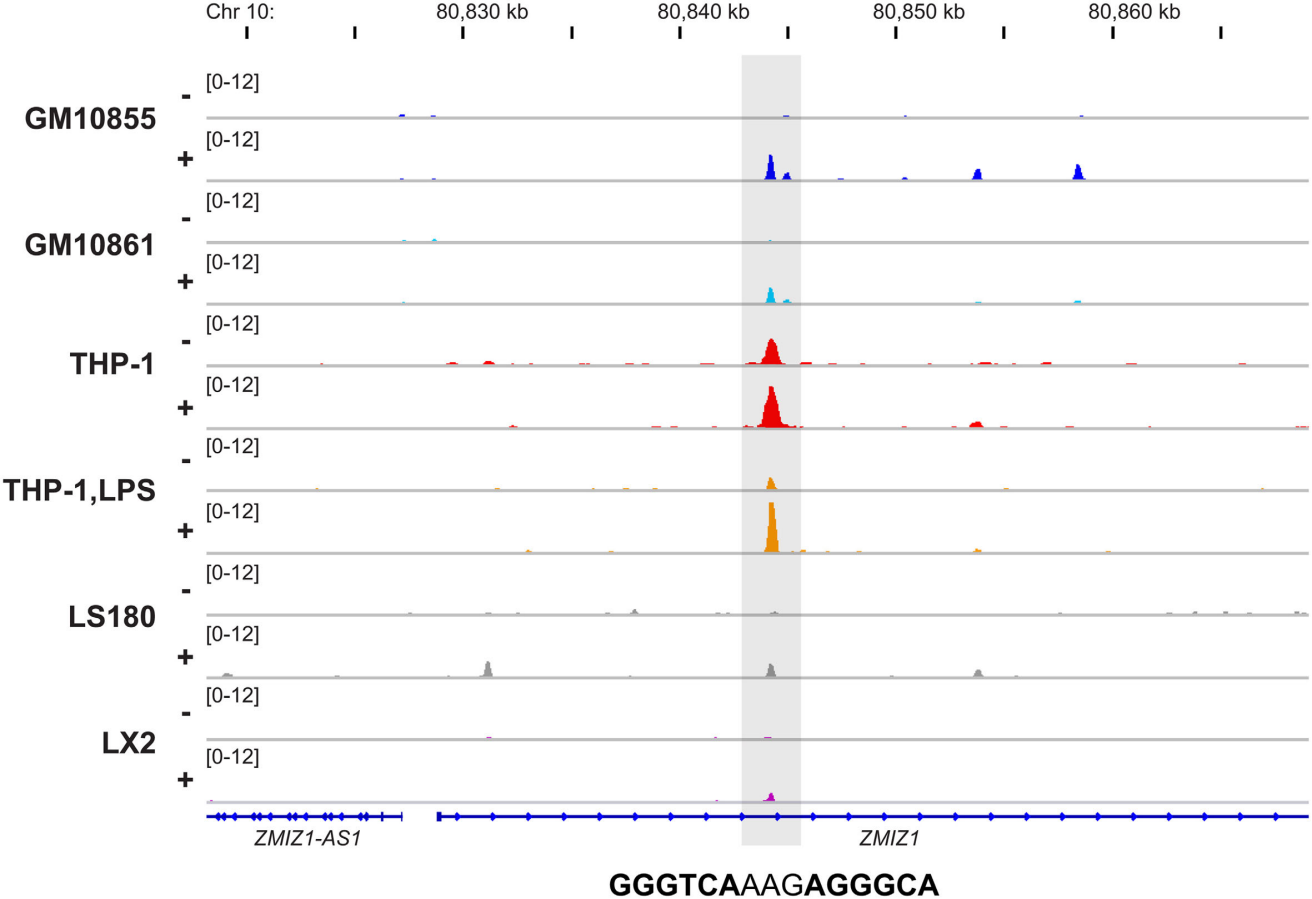
**Conserved genomic VDR binding in six cellular models**. The Integrative Genomics Viewer (IGV) browser (Robinson et al., 2011) was used to visualize the VDR binding site 15.3 kb downstream of the *ZMIZ1* TSS. The peak tracks display data from VDR ChIP-seq datasets from two B cell-like cells (dark and light blue), monocyte-like cells (red), macrophage-like cells (orange), colon cells (gray) and liver cells (violet). The cells were either unstimulated (−) or treated with VDR ligand (+). The gene structures are shown in blue and the sequence of the DR3-type element below the summit of the VDR peak is indicated.

In total, the six VDR ChIP-seq datasets indicated 21,776 non-overlapping VDR binding sites when allowing a distance of up to 250 bp between the peak summits (Tuoresmäki et al., 2014). However, the vast majority of these VDR loci (67%) are unique for one of the analyzed cellular models. In contrast, under the above mentioned conditions only 54 sites are common within all six datasets. In general, this indicates that VDR displays a very individual pattern of cell-specific genomic locations, which overlaps between multiple tissues only at key sites. The VDR binding site of the 1,25(OH)_2_D_3_ target gene *ZMIZ1*, which is located 15.3 kb downstream of the transcription start site (TSS), represents an example of such a locus (Figure 1). In general, the rates of overlaps between the cell types follow roughly their developmental and functional relatedness, i.e., the two lymphoblastoid cell lines, GM10855 and GM10861, or LPS-differentiated and undifferentiated THP-1 cells show more overlapping VDR binding sites than all other comparisons between the VDR ChIP-seq datasets. Moreover, the VDR binding profiles of ligand-stimulated cells matched better than those of unstimulated cells (Tuoresmäki et al., 2014).

Genome-wide studies on VDR binding have changed the view on vitamin D signaling. The few dozens rather well characterized VDR binding sites in less than 10 kb distance to the TSS of 1,25(OH)_2_D_3_ target genes (Haussler et al., 2013), which were known before, were complemented by thousands of additional VDR loci spread over the whole genome. However, the very most of the loci, which were highlighted by ChIP-seq, have not yet been validated by ChIP-qPCR or similar methods (and many will never be confirmed). Some previously known VDR binding sites, such as those controlling the genes *MYC* (Toropainen et al., 2010), *VDR* (Zella et al., 2010), *CCNC* (Sinkkonen et al., 2005), and *ALOX5* (Seuter et al., 2007), could be confirmed by the VDR ChIP-seq datasets. However, for many known 1,25(OH)_2_D_3_ target genes the ChIP-seq data suggest additional or alternative VDR binding sites, many of these being far more distant to the gene's TSS region than previously foreseen. In the past, many of these VDR binding sites had been overlooked due to a focus to only a few kb upstream of the TSS of 1,25(OH)_2_D_3_ target genes. However, in accordance with the results of the ENCODE project (ENCODE-Project-Consortium et al., 2012), VDR binding sites are found with equal probability upstream and downstream of the TSS region of 1,25(OH)_2_D_3_ target genes. In addition, VDR loci in distance of even more than 1 Mb from the gene's TSS are accepted as regulatory sites (more details below).

In summary, there seem to be 1000–10,000 genomic VDR binding sites per cell type. This is far more than the number of primary 1,25(OH)_2_D_3_ target genes, which is in the order of 100-500 per tissue. This even holds true for undifferentiated THP-1 cells, where 774 VDR loci in ligand-stimulated cells are facing 408 statistically significantly up-regulated early 1,25(OH)_2_D_3_ responding genes (Heikkinen et al., 2011). The indicates that some genes are controlled by more than one VDR binding site, i.e., they may have a higher potential to be regulated by 1,25(OH)_2_D_3_ than target genes with only one active VDR locus (more details on the transcriptome-wide response to 1,25(OH)_2_D_3_ in the article by Campbell et al. in this issue).

## Mechanistic insight from VDR ChIP-seq studies

The close to 22,000 non-overlapping VDR peaks, which are indicated by the public ChIP-seq datasets (Tuoresmäki et al., 2014), show rather different characteristics. Despite the rather different total number of reported VDR peaks per cellular model, each of the six ChIP-seq datasets contains an in part overlapping subset of less than 200 sites, where a stimulation with 1,25(OH)_2_D_3_ resulted in a significant increase of VDR binding compared to unstimulated samples. These VDR loci are far more prominent than most of the other sites, for which ligand treatment was either repressive, had no effect or was only minor stimulatory.

Another important parameter for the characterization of a VDR binding site is the presence or absence of a high confidence DR3-type binding site below the summit (±100 bp) of the respective ChIP-seq peak. This can be investigated with the help of binding site screening algorithms, such as provided by HOMER (Heinz et al., 2010). Depending on the threshold settings the software detects binding sites that deviate more or less from the consensus sequence. For example, for a moderate setting of a HOMER score of 7, from the total of 21,776 non-overlapping VDR sites in all six ChIP-seq datasets only 3801 (17.5%) contain a DR3-type sequence. Interestingly, the percentage of DR3-type motifs differs significantly between the datasets and ranges from 38.2% (483 of 1264 sites) in LPS-polarized THP-1 cells via 36.4% (373 of 1023) in undifferentiated THP-1 cells, 28.6% (1062 of 3706) in LS180 cells, 27.8% (611 of 2194) in LX2 cells, 13.0% (909 of 6975) in GM10855 cells to 9.0% (1118 of 12,438) in GM10861 cells. This indicates that the total number of identified VDR binding sites in each cell line inversely correlates with the percentage of peak summits with DR3-type sites. However, when the analysis is restricted to the top 200 VDR sites (based on fold enrichment scoring), for all six ChIP-seq datasets a DR3-like sequence rate of more than 60% is observed, i.e., DR3 motifs are found preferentially at highly ligand responsive VDR loci. In this way, the different VDR ChIP-seq datasets show a very similar relationship between VDR occupancy and DR3 percentage. This suggests that the mechanistic basis for the action of the VDR is independent of the cell type and the total number of identified binding sites.

Transcription factor binding site screening software, such as HOMER, suggests that DR3-type binding sequences are the most abundant sites below the summits of VDR ChIP-seq peaks. However, a significant number of the genomic VDR loci (depending on the dataset 60-90% of all, see above) do not associate with a DR3-type site. This indicates that at these loci VDR uses a different mode of interaction with genomic DNA. This could be either the use of a different heterodimeric binding partner or an indirect binding “backpack” of a DNA-binding transcription factor (Carlberg and Campbell, 2013). In both scenarios the specific DNA binding site would be different to a DR3-type sequence. Interestingly, for the VDR ChIP-seq datasets originating from hematopoietic cells, HOMER indicated binding sites for the transcription factors PU.1 (also called SPI1), ESRRB (also called NR3B2) and GABPA as significantly enriched (Tuoresmäki et al., 2014). PU.1 is well-known as a pioneer factor (Zaret and Carroll, 2011), i.e., as a transcription factor with (i) a high number of genomic binding sites, (ii) a greater binding promiscuity and (iii) higher diversity of interactions. Pioneer factors are the first that bind regulatory genomic regions, such as promoters and enhancers, and interact with chromatin modifying enzymes, in order make the chromatin more accessible for regular transcription factors, such as VDR. At present, a direct protein-protein interaction of VDR with PU.1, ESRRB or GABPA has not been demonstrated, but for the Ets family, to which PU.1 belongs, there were indications for an interaction (Tolon et al., 2000). However, for the pure function as a pioneer factor a direct protein-protein interaction with the “settler factor” is not needed. Moreover, there is older evidence from single gene studies that DNA binding of VDR is modulated by the transcription factors AP1 (Schüle et al., 1990) and RUNX2 (Sierra et al., 2003). In contrast, a genome-wide study on the interaction of VDR with the transcription factor TCF7L2 did not provide any evidence that the latter acts as a pioneer factor for VDR (Meyer et al., 2012).

Below VDR peak summits no dominant non-DR3 binding sequence could be identified. Moreover, the six VDR ChIP-seq datasets differ in the ranking and identity of the non-DR3 sites found below the peaks (Tuoresmäki et al., 2014). This suggests that in total there must be a larger number of VDR partnering proteins. Most likely, these proteins have a cell-specific expression pattern and may explain in part the cell-specific actions of VDR and its natural ligand 1,25(OH)_2_D_3_. Moreover, ChIP-seq datasets have indicated that, in contrast to steroid receptors, VDR binds a number of its genomic targets already in the absence of ligand. These ligand-independent genomic VDR loci have a clearly lower rate of DR3-type sequences than ligand-dependent sites (Heikkinen et al., 2011). In contrast, they associate preferentially with proteins related to gene repression, such as demonstrated for the example of the *CYP27B1* gene (Turunen et al., 2007). This implies that the functional profile of VDR is larger than that of its ligand (Polly et al., 2000) as previously shown for other members of the nuclear receptor superfamily, such as thyroid hormone receptor or liver X receptor (Perissi et al., 2010).

Taken together, all VDR ChIP-seq studies confirm the preferential binding of VDR to DR3-type sequences. However, only one in six of some 22,000 presently known VDR loci within the human genome carry a DR3 site. Thus, there have to be additional mechanisms for the association of VDR with its genomic loci, which may include partnering with presently undefined partner proteins or the tethering to other DNA-binding transcription factors, such as pioneer factors. These should explain some of the cell-specific actions as well as repressive functions of 1,25(OH)_2_D_3_ and the VDR.

## Responses of chromatin to 1,25(OH)_2_D_3_

Histone proteins forming the nucleosome core are DNA-binding proteins but do not show any sequence specificity. Therefore, the complex of nucleosomes and genomic DNA, which is referred to as chromatin, has an intrinsic repressive potential: it prevents access of transcription factors to their genomic targets (Razin, 1998). This provides essential stability to the epigenetic landscape for long-lasting regulatory decisions, such as gene expression in terminally differentiated cells (Mohn and Schubeler, 2009). In contrast, some regions of the epigenome show highly dynamic changes in response to extra- and intracellular signals, such as the activation of VDR by 1,25(OH)_2_D_3_ binding (Talbert and Henikoff, 2006). These changes involve the methylation of genomic DNA and/or reversible post-translational modifications of histone proteins, such as acetylation or deacetylation at exposed lysine residues (Narlikar et al., 2002). Dynamic chromatin modifications change the access to regulatory genomic regions, such as promoter and enhancers, for the binding of transcription factors, i.e., they determine whether at these regions chromatin is open or closed. This can be monitored genome-wide by using the method DNase I hypersensitive sites sequencing (DNase-seq), which highlights genomic regions being most sensitive to cleavage by the enzyme DNase I (Crawford et al., 2006). A very similar technique is Formaldehyde-Assisted Isolation of Regulatory Elements sequencing (FAIRE-seq), which identifies genome-wide accessible DNA regions (Giresi et al., 2007) (more details on the relation of the epigenome and 1,25(OH)_2_D_3_ in the article by Kallay et al. in this issue).

**Figure 2:**
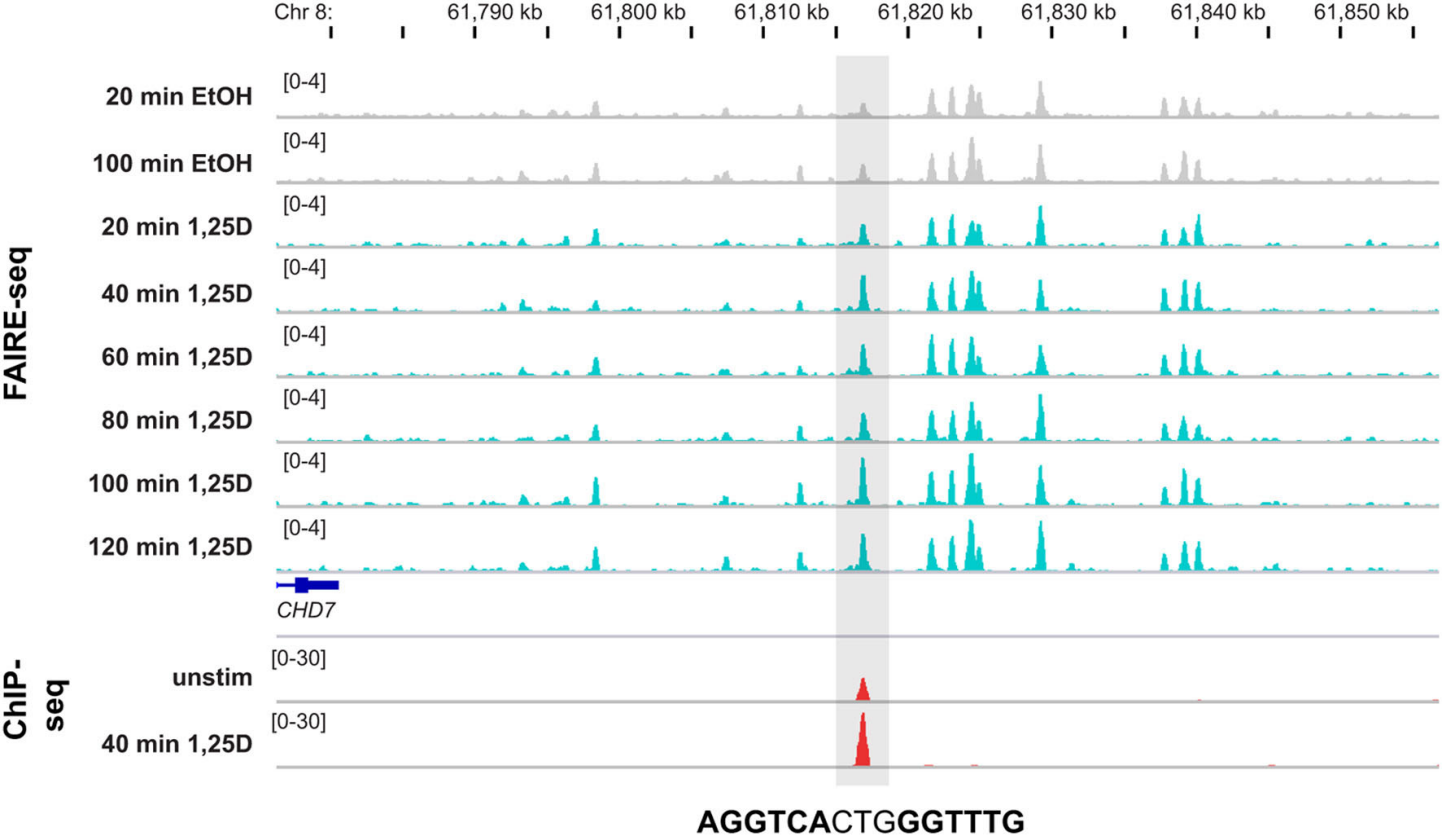
**Genomic view of 1,25(OH)_2_D_3_-dependent chromatin opening**. The IGV browser visualizes the loci of a VDR locus 225 kb downstream of the *CHD7* gene (±40 kb of the peak summit). The peak tracks display data from THP-1 cells: a time course of FAIRE-seq data [gray for EtOH-treated controls and turquoise for 1,25(OH)_2_D_3_ (1,25D) treatments for the indicated time periods] and a VDR ChIP-seq data [red, from unstimulated cells and after 40 min 1,25(OH)_2_D_3_ treatment]. The gene structures are shown in blue and the sequence of the DR3-type element below the summit of the VDR peak is indicated.

At present, the only publically available dataset describing genome-wide effects of 1,25(OH)_2_D_3_ on the epigenome, is a detailed FAIRE-seq time course in THP-1 cells (Seuter et al., 2013). These data demonstrate that some 87% of the more than 1000 VDR binding sites in this cellular model co-localize with open chromatin. Interestingly, at 165 of these VDR loci a strong 1,25(OH)_2_D_3_-dependent increase of chromatin accessibility is found. Importantly, at 66% of these chromatin regions a DR3-type sequence is found, i.e., they overlap with loci, at which VDR binding is enhanced most by 1,25(OH)_2_D_3_ stimulation (Seuter et al., 2013). Moreover, the binding of VDR to its genomic loci is a dynamic process, which takes at least some 2 h to saturate the sites. One example is a site located 225 kb downstream of the TSS of the chromodomain helicase DNA binding protein 7 (*CHD7*) gene (Figure 2). It demonstrates that at the same locus, where a strong ligand-dependent increase of VDR binding is observed, the rate of open chromatin more than doubled already 40 min after incubation of THP-1 cells with 1,25(OH)_2_D_3_. At some 200 additional VDR binding loci the chromatin shows detectable but less prominent response to 1,25(OH)_2_D_3_ treatment, while at the remaining 500 sites the VDR ligand did not affect chromatin accessibility. Accordingly, only at less than 20% of the latter sites DR3-type sequences are found. At many of these sites, VDR binds already in the absence of ligand and may have a different mode of DNA recognition and action (see above).

In summary, at approximately a third of its genome-wide binding loci VDR dynamically controls the epigenetic state of chromatin. At these sites, VDR binding and chromatin opening are tightly interconnected and provide indications for primary 1,25(OH)_2_D_3_ target genes. This allows a better understanding of the 1,25(OH)_2_D_3_ signaling cascade.

## The use of encode data for understanding 1,25(OH)_2_D_3_ signaling

In addition to the above described 1,25(OH)_2_D_3_-triggered chromatin open, in the future there will be much more data available on the interaction of VDR and 1,25(OH)_2_D_3_ with the epigenome. This will include FAIRE-seq and DNase-seq studies in further 1,25(OH)_2_D_3_-responsive tissues as well as investigations on changes of histone modifications and/or co-factor binding in 1,25(OH)_2_D_3_-responsive genomic regions. However, already at present existing genome-wide data on the annotation of the genomes of human, mouse and other species can be used. The best example is the large range of data collected by the ENCODE consortium (ENCODE-Project-Consortium et al., 2012). The core of the ENCODE datasets are publically available ChIP-seq results on approximately 100 transcription factors and 20 histone modifications from more than 100 human cellular systems. From the latter, the human monocytic leukemia cell line K562 is represented with highest number of datasets, while the majority of the other cells has not been studied with the same intensity. At present, ENCODE data describe primarily the basal status of cells, i.e., only in a very few cases a stimulation with hormones, growth factor, cytokines or similar molecules had been performed. Neither data on 1,25(OH)_2_D_3_ stimulations nor VDR ChIP-seq data are comprised in the ENCODE dataset. Nevertheless, the examples shown below will illustrate, how already on this stage ENCODE data are useful for a more detailed understanding of 1,25(OH)_2_D_3_ signaling.

All six VDR ChIP-seq datasets agree with observations of the ENCODE project that (i) transcription factors bind equally likely both up- and downstream of their target gene TSSs and (ii) the likelihood of detecting functional transcription factor binding sites for a given gene decreases by distance from its TSS region (ENCODE-Project-Consortium et al., 2012). This means that, in relation to the TSS of primary 1,25(OH)_2_D_3_ target genes, the distribution of the VDR binding sites has a Gaussian shape. In turn, this suggest that on the same chromosome there would be no threshold distance for the interaction between a VDR binding locus and the TSS of a primary 1,25(OH)_2_D_3_ target gene. However, there are limitations provided by higher-order structures of chromatin.

**Figure 3:**
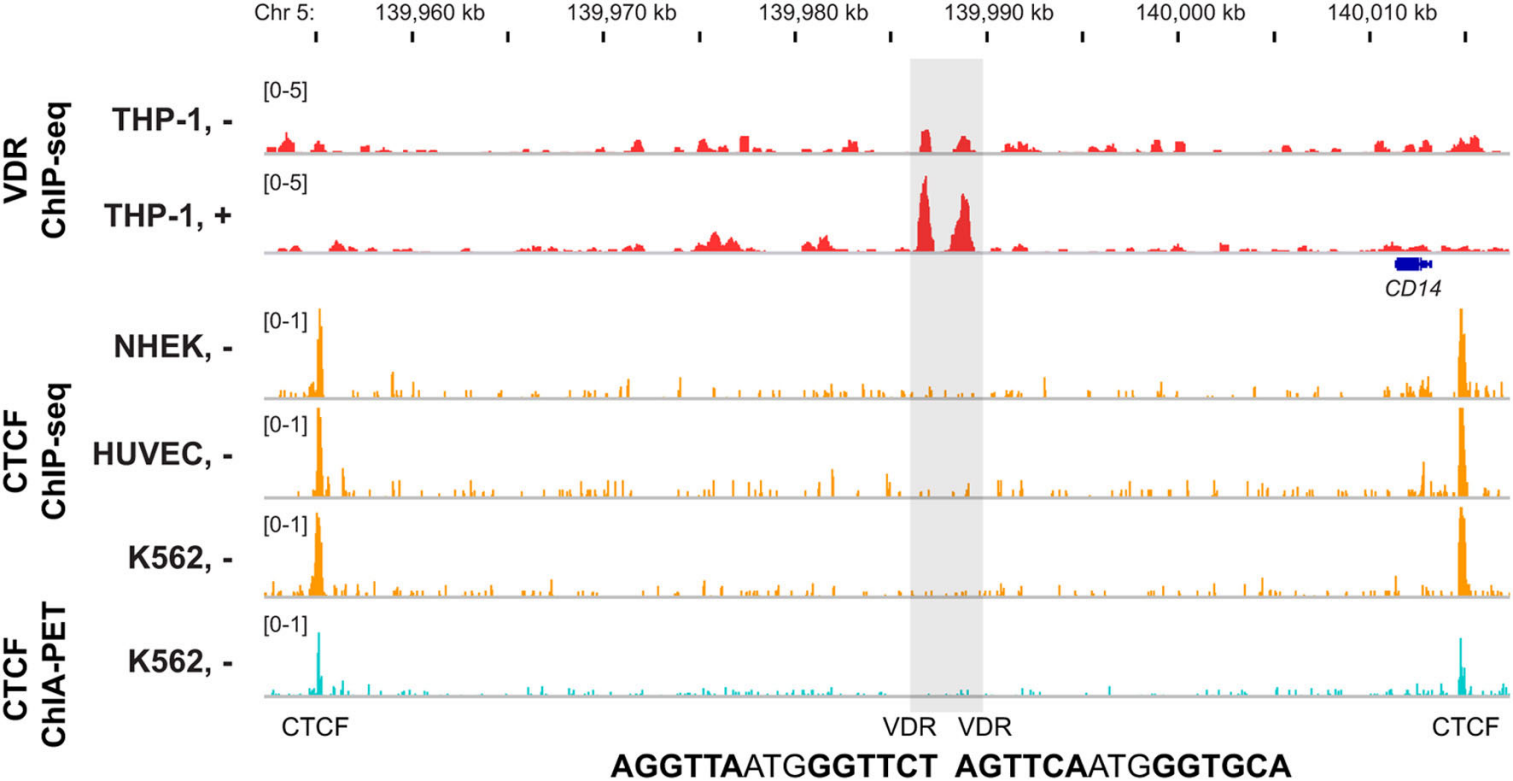
**Chromatin domain containing VDR binding sites**. The IGV browser was used to display the chromatin domain around the *CD14* gene. VDR ChIP-seq data from THP-1 cells [unstimulated (−) and treated for 40 min with 1,25(OH)_2_D_3_ (+), red] are shown in comparison with CTCF ChIP-seq data from the ENCODE cell lines NHEK, HUVEC and K562 (orange) and CTCF ChIA-PET data from K562 cells in the track view (light blue). The gene structures are shown in blue and the sequence of the DR3-type elements below the summits of the VDR peaks are indicated.

Chromatin forms loops (Kadauke and Blobel, 2009), which contribute to many nuclear functions, such as the control of gene expression (Misteli, 2007). Chromatin loops segregate each chromosome into domains, which are separated by an insulator region (Van Bortle and Corces, 2013). Most insulator regions are associated with the highly conserved transcription factor CCCTC-binding factor (CTCF) (Schmidt et al., 2012). Therefore, ChIP-seq data for CTCF binding from multiple human cell lines, such as provided by ENCODE (ENCODE-Project-Consortium et al., 2012), allow a first estimation of the chromatin domain borders (Figure 3). However, only 15–20% of all genomic CTCF binding sites are involved in insulator function. The method chromatin interaction analysis by paired-end tag sequencing (ChIA-PET) (Fullwood et al., 2009) allows an assessment of the 3-dimensional structure of chromatin. When applied for CTCF in K562 cells it mapped more than 120,000 intra-chromosomal, CTCF-mediated chromatin interactions (ENCODE-Project-Consortium et al., 2012). The high conservation of CTCF binding sites allows a reliable extrapolation of the CTCF ChIA-PET data from K562 cells to THP-1 cells, for which VDR ChIP-seq data is available. The combination of both datasets suggests that in THP-1 cells there are some 1600 chromatin domains, which contain at least one VDR binding site (Seuter et al., 2014). When the TSS region of a gene is within one of these chromatin regions, it may be a primary 1,25(OH)_2_D_3_ target. In case of the *CD14* gene, CTCF ChIA-PET data from K562 cells defined a chromatin domain spanning from 1.5 kb upstream to 57 kb downstream of the gene's TSS (Figure 3). This domain spans over the whole *CD14* gene and comprises two VDR binding sites 24 and 26 kb downstream of the gene's TSS. This provides a straightforward gene regulatory scenario explaining (i) the primary response of *CD14* to 1,25(OH)_2_D_3_ and (ii) why the neighboring genes of *CD14* do not respond to VDR ligand treatment.

The chromatin domain of the *CD14* gene is with a size of less than 60 kb rather small (Figure 3). In contrast, one of the largest VDR-containing chromatin domains spans over 2.3 Mb of chromosome 8 and combines the *MYC* gene with four VDR binding sites, of which the most prominent is 1215 kb of the gene's TSS (Ryynänen et al., 2013). This suggests that under the condition of sufficiently large chromatin domains, gene regulation by VDR can be over a distance of more than 1 Mb.

At present, there is no VDR ChIA-PET data available but will come in the future. However, via the ENCODE experiment matrix (http://encodeproject.org/ENCODE/dataMatrix/encodeDataMatrixHuman.html) there is access to ChIA-PET data for RNA polymerase II in HeLa human cervix carcinoma and MCF-7 human breast carcinoma and for estrogen receptor α in MCF-7 cells. The latter may be of special interests for the breast cancer field.

Taken together, public ENCODE data are important tools, which can be used in combination with genome-wide data on VDR for an extrapolation on the 3-dimensional organization of gene regulation by 1,25(OH)_2_D_3_.

## 1,25(OH)_2_D_3_ target genes as biomarkers for the vitamin D status of human individuals

In contrast to a number of other nuclear receptor ligands, such as cortisol or estrogen, the endocrinology of 1,25(OH)_2_D_3_ does not imply any fast changes (Deluca, 2004; Norman, 2008). Under normal circumstances, either the production in UV-B exposed skin or the intake of from diet or supplements should provide sufficient amounts of vitamin D_3_, in order to achieve optimal serum 25(OH)D_3_ concentrations. The latter vitamin D metabolite is the widely accepted indicator of the vitamin D_3_ status of the human body (Hollis, 2005). The serum 25(OH)D_3_ concentrations change only in the order of weeks and months, such as the result of seasonal variations in sun exposure (Virtanen et al., 2011). This indicates that stimulation experiments with 1,25(OH)_2_D_3_ over a few hours, as performed in *in vitro* experiments, do not represent the physiological reality. In contrast, the effects of more long-lasting changes of serum 25(OH)D_3_ concentrations should be considered. On a genome-wide level, this was investigated first with primary T cells isolated from nine human individuals with variant serum 25(OH)D_3_ concentrations (Handel et al., 2013). The number of the observed VDR ChIP-seq peaks, which varied between 200 and more than 7000, correlated with the 25(OH)D_3_ levels of the individuals, i.e., the higher the circulating 25(OH)D_3_ concentrations, the more VDR loci were identified in T cells. Unfortunately, the raw data of this study is not available, i.e., a harmonized re-analysis in comparison with other published VDR ChIP-seq data cannot be performed. However, from the 14,044 unique VDR peaks reported for the sum of the nine individuals, only 442 (3.1%) associated with a DR3-type sequence (based on HOMER score 7 settings).

Serum 25(OH)D_3_ concentrations vary widely from person to person based on (i) varied diet and sun exposure, (ii) different age and/or level of adiposity and (iii) genetic and epigenetic variations (Engelman et al., 2008; Orton et al., 2008; Snellman et al., 2009). The Institute of Medicine recommends a serum 25(OH)D_3_ level of 50 nM (Institute-of-Medicine, 2011), but it is under debate, whether this is sufficient for every individual (Holick, 2007). In fact, a substantial proportion of the world's population could be considered as vitamin D deficient. This condition may accelerate age-related bone loss and morbidity from falls and fractures. In addition, vitamin D insufficiency is associated with a number of diseases, such as cancer, autoimmune disorders and all components of the metabolic syndrome (more details in the article by Bendik et al. in this issue).

This important medical problem guided to the question, whether an insight into the genome- and transcriptome-wide actions of VDR and 1,25(OH)_2_D_3_ can help in a more accurate evaluation of the human individual's responsiveness to, and needs for, vitamin D. A first approach in this direction was done by studying peripheral blood mononuclear cells (PBMCs) and adipose tissue biopsies from 71 elderly, pre-diabetic individuals, which participated in a 5-month vitamin D_3_ intervention trial (VitDmet) during Finnish winter (Carlberg et al., 2013). The changes in the mRNA expression of the primary 1,25(OH)_2_D_3_ target genes *CD14* and thrombomodulin (*THBD*), which had been identified in a recent comparative study as most reliable biomarkers (Standahl Olsen et al., 2013), in both PBMCs and fat samples were correlated with the alterations in the serum 25(OH)D_3_ levels of the 71 individuals. Interestingly, only for a subset of individuals significant correlations between the up-regulation of both genes and the intervention-induced raise in serum 25(OH)D_3_ concentrations were obtained. This suggests that, on a molecular level, not all study participants benefited from the vitamin D_3_ supplementation, because (i) they had already reached their individual optimal vitamin D status before the start of the intervention, (ii) they carry a genetic polymorphism making them less responsive to vitamin D_3_ or (iii) other undefined reasons (Carlberg et al., 2013). Interestingly, the categorization of the human individuals by their vitamin D responsiveness unmasked a negative correlation between changes in serum concentrations of 25(OH)D_3_ and the inflammation marker interleukin 6, i.e., the more responsive the study participants were to vitamin D_3_ supplementation, the lower was their inflammatory status. At present, a number of other primary 1,25(OH)_2_D_3_ target genes, which were highlighted in the comparison of VDR ChIP-seq data, are evaluated for their potential to serve as even better biomarkers for the vitamin D status of human individuals than *CD14* and *THBD*.

In summary, vitamin D deficiency may negatively contribute to a number of diseases. Genome-wide insight led to the use of mRNA expression changes of the genes *CD14* and *THBD* as biomarkers for a molecular evaluation of vitamin D_3_ supplementation studies. The results allow a classification of human individuals based on their responsiveness to vitamin D_3_.

## Conclusions

Genome-wide data on (i) the location of transcription factor binding sites in living cells, (ii) histone modifications and (iii) accessible chromatin, such as provided by ChIP-seq, DNase-seq and FAIRE-seq studies, have significantly changed the view on, and the understanding of the regulation of the entirety of the genes of our genome. This applies also to the transcription factor VDR, for which at present ChIP-seq data from six human cell lines and the T cells of nine human individuals are available. The abovementioned modern genome-wide techniques allow a more unbiased identification of transcription factor binding sites compared to previous studies, which were mostly focused on regions a few kb upstream of a primary 1,25(OH)_2_D_3_ target gene. VDR binding loci have now been shown to be localized equally likely up- and downstream of TSS regions in distances of even more than 1 Mb.

Genome-wide studies have confirmed DR3-type sequences as the preferential binding sites for VDR (most likely as a heterodimer with RXR), but only one in seven of the close to 20,000 known VDR binding loci carry such a motif. This is unanimously observed in all investigated cellular models. Therefore, there must be other types of binding motifs and partnering proteins that attract VDR to its genomic targets. These presently poorly understood alternative binding modes may explain some of VDR's function in the (trans)repression of its target genes. Moreover, the VDR cistrome seems to be largely cell-specific with only some 50 loci overlapping in all investigated models. However, these conserved sites could be fundamental entry ports of VDR to the human genome, which may serve as the unified core of the various pleiotropic functions of 1,25(OH)_2_D_3_.

The 22,000 detected VDR binding loci within six cell lines as well as the 14,000 peaks found in primary T cells from nine human individuals may be far more than what is needed to control the physiological actions of 1,25(OH)_2_D_3_, i.e., many sites may represent rather “noise” than having a specific function. Therefore, different approaches to categorize VDR loci are useful. It turned out that VDR binding sites that (i) carry a DR3-type sequence, (ii) show ligand-stimulated VDR association, (iii) co-locate with ligand-induced chromatin opening and (iv) are conserved between several cellular systems may play a more important role in mediating the functions of 1,25(OH)_2_D_3_ than the vast majority of other VDR sites that lack most of these properties. Therefore, for a cellular system of interest, the combination of (i) a genome-wide assessment of open chromatin by DNase-seq or FAIRE-seq, (ii) the monitoring of genomic VDR loci by ChIP-seq and (iii) a screening for DR3-type sequences below the peak summits is an efficient tool for the prediction and identification of primary 1,25(OH)_2_D_3_ target genes.

## Future perspectives

Although historically 1,25(OH)_2_D_3_ was understood to be a hormone controlling calcium homeostasis and bone formation, to date the genome-wide the actions of VDR are best understood and monitored in cells of the hematopoietic system. This emphasizes the impact of 1,25(OH)_2_D_3_ on the function of innate and adaptive immunity. There are first indications that the core actions of 1,25(OH)_2_D_3_ and its receptor VDR can be extrapolated from hematopoietic cells to other tissues and cell types of the human body (Carlberg et al., 2013). If this holds true, the vitamin D status and responsiveness of a human individual can be derived from the response of, for example, PBMCs. Technically, various types of leukocytes can be collected far easier from blood samples than any other tissue biopsy. Like a glucose tolerance test is used to monitor the functionality of the carbohydrate metabolism of an individual, there may be in the future a higher dose vitamin D_3_ challenge test, where the (epi)genomic and transcriptomic profiles of leukocytes before and after supplementation are measured. Routine measurements of healthy individuals may also in the future more likely be based on a few selected biomarkers, such as *CD14* and *THBD*, while more complex scenarios in disease settings, such as cancer or autoimmune diseases, will be assessed genome- or transcriptome-wide. In the same way, basic research on 1,25(OH)_2_D_3_ and VDR will shift more and more from cell culture models to primary tissues and cell types and will eventually reach the single cell level.

### Conflict of interest statement

The authors declare that the research was conducted in the absence of any commercial or financial relationships that could be construed as a potential conflict of interest.

## Abbreviations

1,25(OH)_2_D_3_: 1α,25-dihydroxyvitamin D_3_
25(OH)D_3_: 25-hydroxyvitamin D_3_
ALOX5: arachidonate 5-lipoxygenase
CBS: cystathionine β-synthase
CCNC: cyclin C
CDKN1A: cyclin-dependent kinase inhibitor 1A
CHD7: chromodomain helicase DNA binding protein 7
ChIA-PET: chromatin interaction analysis by paired-end tag sequencing
ChIP: chromatin immunoprecipitation
ChIP-seq: ChIP coupled with massive parallel sequencing
CTCF: CCCTC-binding factor
CYP: cytochrome P450
DNase-seq: DNase I hypersensitivity sites sequencing
DR3: direct repeat spaced by 3 nucleotides
FAIRE-seq: formaldehyde-assisted isolation of regulatory elements sequencing
IGV: Integrative Genomics Viewer
LPS: lipopolysaccharide
LRP5: low density lipoprotein receptor-related protein 5
MYC: v-myc avian myelocytomatosis viral oncogene homolog
PBMC: peripheral blood mononuclear cell
RXR: retinoid X receptor
THBD: thrombomodulin
TNFSF11: tumor necrosis factor (ligand) superfamily, member 11
TRPV6: transient receptor potential cation channel subfamily V, member 6
TSS: transcription start site
VDR: vitamin D receptor
ZMIZ1: zinc finger, MIZ-type containing 1.
